# Dataset of mRNA levels for dopaminergic receptors, adrenoceptors and tyrosine hydroxylase in lymphocytes from subjects with clinically isolated syndromes

**DOI:** 10.1016/j.dib.2016.08.067

**Published:** 2016-09-17

**Authors:** Marco Cosentino, Mauro Zaffaroni, Massimiliano Legnaro, Raffaella Bombelli, Laura Schembri, Damiano Baroncini, Anna Bianchi, Raffaella Clerici, Mario Guidotti, Paola Banfi, Giorgio Bono, Franca Marino

**Affiliations:** a Center of Research in Medical Pharmacology, University of Insubria, Varese, Italy; b Multiple Sclerosis Center, Hospital ׳S. Antonio Abate׳, Gallarate, VA, Italy; c Neurological Department, Valduce Hospital, Como, Italy; d Department of Biotechnology and Life Sciences, University of Insubria, Varese, Italy

**Keywords:** Clinically isolated syndrome, Multiple sclerosis, mRNA levels, Dopaminergic receptors, Adrenoceptors, Peripheral blood mononuclear cells, CD4+ T effector lymphocytes, CD4+ T regulatory lymphocytes

## Abstract

This data article presents a dataset of mRNA levels for dopaminergic receptors, adrenoceptors and for tyrosine hydoxylase, the rate-limiting enzyme in the synthesis of catecholamines, in peripheral blood mononuclear cells as well as in CD4+ T effector and regulatory cells from subjects with clinically isolated syndromes (CIS), which is a first episode of neurological disturbance(s) suggestive of multiple sclerosis. CIS subjects are divided into two groups according to their eventual progression, after 12 months from CIS, to clinically established multiple sclerosis. The data reported are related to the article entitled "Dopaminergic receptors and adrenoceptors in circulating lymphocytes as putative biomarkers for the early onset and progression of multiple sclerosis" (M. Cosentino, M. Zaffaroni, M. Legnaro, R. Bombelli, L. Schembri, D. Baroncini, A. Bianchi, R. Clerici, M. Guidotti, P. Banfi, G. Bono, F. Marino, 2016) [1].

**Specifications Table**

**Table:** 

Subject area	*Medicine*
More specific subject area	*Neurology, Immunology, Neuroimmunology*
Type of data	*Tables*
How data was acquired	*Real-time PCR, ABI PRISM*® *7000 System (Applied Biosystems, Life Technologies Corporation, USA), data statistical analysis (GraphPad Prism version 5.00 for Windows, GraphPad Software, San Diego California USA,*www.graphpad.com*)*
Data format	*Analyzed*
Experimental factors	*Peripheral blood mononuclear cells (PBMC) isolated by gradient centrifugation from whole blood of subjects with clinically isolated syndromes (CIS), and cultured for 48 h alone or with PHA 10 μg/ml. A sample of freshly isolated PBMC was used to isolate CD4+ T effector (Teff) and regulatory (Treg) cells by means of immunomagnetic sorting.*
Experimental features	*Real-time PCR analysis of mRNA levels of dopaminergic receptors, adrenoceptors and tyrosine hydroxylase mRNA levels, following total RNA extraction by PerfectPure™ RNA Cell & Tissue kit (5Prime, Milano, Italy), reverse transcription by a random primer and a high-capacity cDNA RT kit (Applied Biosystems, Life Technologies Corporation, USA), and cDNA amplification by TaqMan® Universal PCR Master Mix (Applied Biosystems), using the TaqMan Gene Expression Assay.*
Data source location	*Varese, Gallarate, Como (Italy)*
Data accessibility	*Data is within this article*

**Value of the data**

• These data provide the profile of expression of dopaminergic receptors, adrenoceptors and tyrosine hydroxylase genes in circulating lymphocytes of subjects with clinically isolated syndromes (CIS).
• The data are of value for further experiments on the mechanistic role of dopaminergic and adrenergic pathways in circulating lymphocytes during CIS and multiple sclerosis (MS).
• The data give a basis for longitudinal, prospective clinical studies aimed at validating dopaminergic receptors and/or adrenoceptors gene expression in lymphocytes as early markers of CIS progressing to MS.

## 1 Data

Enclosed are data regarding mRNA levels for dopaminergic receptors (DR), adrenoceptors (AR) and tyrosine hydroxylase (TH), the rate-limiting enzyme in the synthesis of catecholamines, found in peripheral blood mononuclear cells (PBMC) (Table 1, Table 2) and in CD4+ T effector (Teff) cells (Table 3) and regulatory (Treg) cells (Table 4) from subjects with clinically isolated syndromes (CIS), which is a first, usually recovering, episode of neurological disturbance(s) suggestive of multiple sclerosis (MS). Each table provides a comparison between subjects who, after 12 months from CIS, did not progress or progressed to clinically established MS. For further information and discussion about the interpretation and implications of DR, AR and TH mRNA levels in lymphocytes of CIS subjects, please refer to the article [1].

## 2 Experimental design, materials and methods

### 2.1 PBMC isolation and culture

Cells were obtained from venous blood of CIS subjects enrolled at the Centre for research on Multiple Sclerosis, Ospedale S. Antonio Abate of Gallarate (VA) (Investigator in charge: Mauro Zaffaroni), at the Neurology Unit of the “Ospedale di Circolo e Fondazione Macchi”, University of Insubria - School of Medicine of Varese (Investigator in charge: Giorgio Bono), and at the Neurological Department, Valduce Hospital, Como (Investigator in charge: Mario Guidotti). Inclusion and exclusion criteria for selection and enrollement of CIS subjects, as well as criteria to define conversion of CIS to MS are detailed elsewhere [1]. Approval of the protocol was obtained from the Ethics Committee of the Ospedale S. Antonio Abate of Gallarate (VA), and all the participants provided a written informed consent.

PBMC were isolated from whole blood by using Ficoll–Paque Plus density gradient centrifugation, using standard procedures [2]. PBMC were finally cultured in RPMI 1640/10% heath-inactivated fetal bovine serum, added with 2 mM glutamine and 100 U/ml penicillin/streptomycin, at the concentration of 1×10^6^ cells/ml, at 37 °C in a moist atmosphere of 5% CO_2_. Cells were cultured for 48 h, alone or in the presence of PHA 10 μg/ml, a concentration which was previously shown to be optimal to trigger mRNA expression of TH [3]. PBMC were finally harvested and assayed for DR, AR and TH mRNA expression by means of real-time PCR.

### 2.2 Preparation of Teff and Treg

Immunomagnetic sorting of Treg and Teff from freshly isolated PBMC was performed by using the Dynal CD4+CD25+ Treg Kit (Dynal, Oslo, Norway), as previously described [4]. Treg and Teff were directly assayed for DR, AR and TH mRNA expression by means of real-time PCR.

### 2.3 Real-time PCR

Extraction of total RNA was performed with PerfectPure™ RNA Cell & Tissue kit (5Prime, Milano, Italy). RNA was then reverse-transcribed to cDNA using a random primer, high-capacity cDNA RT kit (Applied Biosystems, Life Technologies Corporation, USA), and finally amplified by *Ta*qMan® Universal PCR Master Mix (Applied Biosystems), using the *Taq*Man Gene Expression Assay (Table 5). Assayed of cDNA was accomplished on an ABI PRISM® 7000 System (Applied Biosystems). Gene expression levels were finally expressed as 2^−*ΔCt*^ where *ΔCt=*[*Ct* (sample)−*Ct* (housekeeping gene)], and normalized to 18S cDNA, using the AB Prism 7000 SDS software™. Annealing temperature was 60 °C for all the genes.

### 2.4 Statistics

Data are reported as means±standard deviation (SD). The D׳Agostino & Pearson normality test was used to assess the distribution of values. The two-tailed Student׳s *t* test for unpaired data or the Mann–Whitney test for continuous variables were used to assess differences between groups. Calculations were performed using a commercial software (GraphPad Prism version 5.00 for Windows, GraphPad Software, San Diego California USA, www.graphpad.com).

## Conflict of Interest

All the authors declare that they have no conflict of interest.

## Figures and Tables

**Table 1:** Levels of DR, AR and TH mRNA in resting PBMC from CIS subjects who, after 12 months from CIS, did not convert (CISnc) or converted (CISc) to clinically established MS. Levels of mRNA are expressed as 2^−*ΔCt*^×10^7^.

**Gene**	**CISnc**	**CISc**	**Ratio CISc/CISnc**	***P***
TH	0.544±0.469	0.585±0.643	1.075	0.878
DRD2^a^	0.062±0.010	0.083±0.035	1.336	0.219
DRD3	6.288±2.273	9.868±4.232	1.569	0.041
DRD5	81.073±134.865	46.128±42.210	0.569	0.390
ADRA1A	0.076±0.034	0.082±0.019	1.077	0.618
ADRA1B	0.991±0.702	1.418±0.806	1.431	0.232
ADRA1D	40.271±10.107	43.439±13.895	1.079	0.583
ADRA2A	0.075±0.046	0.116±0.070	1.559	0.151
ADRA2B	undetected	undetected	n/a	n/a
ADRA2C	0.055±0.023	0.053±0.033	0.961	0.873
ADRB1	0.554±0.329	0.342±0.180	0.617	0.070
ADRB2^b^	0.659±0.582	1.180±1.781	1.790	0.438
ADRB3	0.149±0.084	0.186±0.079	1.244	0.329

Notes:

n/a=not applicable.

a =levels of mRNA below detection limits in 3 CISnc and 3 CISc subjects;

b =data from sample of one CISc subject excluded from the analysis due to assay failure.

**Table 2:** Levels of DR, AR and TH mRNA in PHA-stimulated PBMC from CIS subjects who, after 12 months from CIS, did not convert (CISnc) or converted (CISc) to clinically established MS. Levels of mRNA are expressed as 2^−*ΔCt*^×10^7^.

**Gene**	**CISnc**	**CISc**	**Ratio CISc/CISnc**	***P***
TH	17.349±19.878	21.587±23.140	1.244	0.673
DRD2	0.111±0.044	0.122±0.096	1.096	0.773
DRD3	212.251±102.955	227.008±146.229	1.070	0.806
DRD5	615.891±755.387	447.890±448.168	0.727	0.527
ADRA1A	0.176±0.034	0.270±0.116	1.540	0.052
ADRA1B	149.347±204.884	153.014±225.484	1.025	0.971
ADRA1D	212.433±152.730	199.297±132.607	0.938	0.837
ADRA2A	0.634±0.484	1.431±0.957	2.257	0.043
ADRA2B^a^	0.034±0.012	0.051±0.007	1.493	0.046
ADRA2C	0.183±0.169	0.202±0.130	1.105	0.771
ADRB1	1.520±0.794	1.308±0.668	0.861	0.519
ADRB2^b^	6.156±2.782	8.788±4.242	1.428	0.141
ADRB3	0.602±0.369	0.659±0.295	1.095	0.698

Notes:

a =levels of mRNA below detection limits in 5 CISnc and 8 CISc subjects;

b =data from sample of one CISc subject excluded from the analysis due to assay failure.

**Table 3:** Levels of DR, AR and TH mRNA in Teff from CIS subjects who, after 12 months from CIS, did not convert (CISnc) or converted (CISc) to clinically established MS. Levels of mRNA are expressed as 2^−*ΔCt*^×10^7^.

**Gene**	**CISnc**	**CISc**	**Ratio CISc/CISnc**	***P***
TH	0.932±0.667	1.514±1.680	1.625	0.367
DRD2^a^	0.061±0.012	0.091±0.037	1.495	0.083
DRD3	71.274±40.127	160.645±224.222	2.254	0.283
DRD5	38.986±32.811	45.656±25.700	1.171	0.617
ADRA1A	0.104±0.027	0.136±0.038	1.303	0.056
ADRA1B	23.090±20.545	40.727±30.437	1.764	0.170
ADRA1D	121.840±68.792	155.881±73.368	1.279	0.312
ADRA2A	0.182±0.116	0.229±0.134	1.263	0.421
ADRA2B^a^	undetected	undetected	n/a	n/a
ADRA2C	0.055±0.034	0.085±0.047	1.547	0.138
ADRB1	0.894±0.411	0.801±0.581	0.896	0.700
ADRB2	5.479±2.738	5.379±4.585	0.982	0.957
ADRB3	0.226±0.140	0.243±0.122	1.076	0.797

Notes:

n/a=not applicable.

a =levels of mRNA below detection limits in 2 CISnc and 3 CISc subjects.

**Table 4:** Levels of DR, AR and TH mRNA in Treg from CIS subjects who, after 12 months from CIS, did not convert (CISnc) or converted (CISc) to clinically established MS. Levels of mRNA are expressed as 2^−*ΔCt*^×10^7^.

**Gene**	**CISnc**	**CISc**	**Ratio CISc/CISnc**	***P***
TH	6.239±5.575	6.029±5.332	0.966	0.935
DRD2^a^	0.098±0.044	0.162±0.083	1.657	0.090
DRD3^b^	524.542±320.649	675.012±505.025	1.287	0.470
DRD5	179.094±96.190	271.145±87.095	1.514	0.044
ADRA1A	0.178±0.084	0.228±0.096	1.277	0.260
ADRA1B	539.118±634.164	685.947±919.180	1.272	0.703
ADRA1D	273.060±162.944	431.602±273.593	1.581	0.164
ADRA2A	0.533±0.401	0.708±0.576	1.329	0.471
ADRA2B^a^	undetected	undetected	n/a	n/a
ADRA2C	0.147±0.152	0.168±0.098	1.139	0.724
ADRB1	2.299±2.183	1.284±0.734	0.558	0.166
ADRB2	13.974±9.163	15.322±9.322	1.096	0.758
ADRB3	0.402±0.228	0.470±0.240	1.170	0.583

Notes:

n/a=not applicable.

a =levels of mRNA below detection limits in 1 CISnc and 3 CISc subjects;

b =data from sample of one CISc subject excluded from the analysis due to assay failure.

**Table 5:** Real-Time PCR conditions.

**Gene**	**UniGene ID**	**Interrogated sequence***RefSeq/GenBank mRNA*	**Protein**	**Exon boundary**	**Assay location**	**Amplicon length**	**Efficiency** (%)
TH	Hs.435609	NM_199292.2	NP_954986.2	3–4	424–422	63	94.5
DRD2	Hs.73893	NM_000795.3	NP_000786.1	2–3	524	64	100.0
DRD3	Hs.121478	NM_033663.3	NP_387512.3	3–4	809–725	73	97.6
DRD5	Hs.380681	NM_000798.4	NP_000789.1	1–1	1092–744	88	110.2
ADRA1A	Hs. 709175	NM_033302.2	NP_150645.2	1–2	1324	112	100.0
ADRA1B	Hs. 368632	NM_000679.3	NP_000670.1	1–2	1126	61	100.0
ADRA1D	Hs. 557	NM_000678.3	NP_000669.1	1–2	1166	68	100.1
ADRA2A	Hs. 249159	NM_000681.3	NP_000672.3	1–1	1960	116	101.0
ADRA2B	Hs. 247686	NM_000682.5	NP_000673.2	1–1	823	117	100.0
ADRA2C	Hs. 123022	NM_000683.3	NP_000674.2	1–1	646	93	99.1
ADRB1	Hs. 99913	NM_000684.2	NP_000675.1	1–1	863	79	99.0
ADRB2	Hs. 2551	NM_000024.5	NP_000015.1	1–1	778	65	100.0
ADRB3	Hs. 2549	NM_000025.2	NP_000016.1	1–2	1401	65	99.9
18S rRNA	X03205.1	N.A.	N.A.	N.A.	N.A.	187	98.8
