# Supplementary material for: Dataset of mRNA levels for dopaminergic receptors, adrenoceptors and tyrosine hydroxylase in lymphocytes from subjects with clinically isolated syndromes
Source: Data Brief. 2016 Sep 17;9:376–81. doi: 10.1016/j.dib.2016.08.067 (PMC5035338; doi:10.1016/j.dib.2016.08.067)
Supplement: Supplementary file 1 — Supplementary material [file mmc1.docx]

**DIB-D-16-00557R1**

**Title: DATASET OF mRNA LEVELS FOR DOPAMINERGIC RECEPTORS, ADRENOCEPTORS AND TYROSINE HYDROXYLASE IN LYMPHOCYTES FROM SUBJECTS WITH CLINICALLY ISOLATED SYNDROMES**

**Authors:** Marco Cosentino, Mauro Zaffaroni, Massimiliano Legnaro, Raffaella Bombelli, Laura Schembri, Damiano Baroncini, Anna Bianchi, Raffaella Clerici, Mario Guidotti, Paola Banfi, Giorgio Bono, Franca Marino

**Conflict of Interest**

All the authors declare that they have no conflict of interest.

This sentence has been also included in the manuscript, before the Reference section. Please note that no Conflict of Interest form could be downloaded from the DIB website.
